# Public and Commercial Medical Insurance Enrollment Rates of Rural-to-Urban Migrants in China

**DOI:** 10.3389/fpubh.2021.749330

**Published:** 2021-11-30

**Authors:** Senhu Wang, Anran Liu, Wei Guo

**Affiliations:** ^1^Department of Sociology, National University of Singapore, Singapore, Singapore; ^2^School of Agricultural Economics and Rural Development, Renmin University of China, Beijing, China; ^3^Department of Social Work and Social Policy, School of Social and Behavioral Sciences, Nanjing University, Nanjing, China; ^4^The Centre for Asia-Pacific Development Studies, Nanjing University, Nanjing, China

**Keywords:** public medical insurance, commercial medical insurance, rural-to- urban migrants, China, public health

## Abstract

**Objectives:** Large-scale rural-to-urban migration of China has provoked heated discussion about the health of migrants and whether they have equal access to the health resources. This article aimed to compare the public and commercial medical insurance enrollment rates between temporary, permanent migrants and urban natives.

**Methods:** Average marginal effects (AME) of the weighted logistic regression models using 2017 China General Social Survey from 2,068 urban natives, 1,285 temporary migrants, and 1,295 permanent migrants.

**Results:** After controlling for the demographic and socio-economic characteristics, our results show that while the temporary and permanent migrants have a similar public insurance enrollment rate compared with the urban natives, both temporary and permanent migrants have significantly lower commercial insurance enrollment rates (7.5 and 5.3%, respectively) compared with the urban natives.

**Conclusions:** The results highlight significant institutional barriers preventing the temporary migrants from gaining access to public medical insurance and the adverse impact of disadvantaged socio-economic backgrounds on the access of temporary migrants to both public and commercial insurance.

## Introduction

### Policy Background

Over the last several decades, the socio-economic transformation of China has led to rapid urbanization as well as large-scale rural-to-urban migration. According to the recent official statistics of the government, there are 230 million people (most of them are rural-to-urban migrants) who reside in a place other than their home town, accounting for around 17% of the total population (1). The high level of population mobility in China has stimulated heated discussion about the health condition of rural migrants (2). It is argued that while the rural migrants of China are healthier than urban natives and their non-migrant counterparts upon their initial arrival in urban areas, their health condition worsens the longer they stay in the host cities (3, 4). For example, research shows that compared with the urban natives, the rural migrants have a higher risk of contracting infectious diseases (e.g. tuberculosis, Hepatitis, and sexually transmitted diseases) (5–7), are more likely to suffer from the occupation related injuries (e.g., chemical poisoning and machinery accidents) (8), and fare worse on indicators of maternal and infant health (9).

Given the large population size of rural migrants in China and their relatively poor health, providing appropriate medical insurance and services has become an important priority of the national health strategy (10). It is widely argued that medical insurance enrollment plays an important role in improving the health status of people as it could not only make high-quality healthcare more affordable for people from economically disadvantaged backgrounds, but also help them to obtain more timely medical care (11, 12). Given the benefits of medical insurance, the Chinese government like many other countries has initiated national medical reform by first introducing the Urban Employee-based Basic Medical Insurance (UEBMI) in 1998. The UEBMI is financed jointly by urban employers and employees' wage, depending on local UEBMI policy (2, 13). Subsequently, the government has introduced the New Rural Cooperative Medical System (NRCMS) in 2003 and the Urban Resident-based Basic Medical Insurance (URBMI) in 2007. While NRCMS targets at all the rural residents, URBMI aims to cover all the urban unemployed residents. For both insurance schemes, about 70–80% of funding comes from the government with the rest 20–30% from the urban and rural residents (11, 13). It is claimed that the three insurance schemes i.e., UEBMI, URBMI, and NRCMS can provide public medical insurance to all the residents of China (12).

Recent research, however, argues that the rural migrants of China often encounter a variety of barriers to access to public and commercial medical insurance (14–20). The Chinese household registration (i.e., hukou) system is widely regarded as an important institutional barrier for the rural migrants to have access to public medical insurance (12, 21). The hukou system was established since the 1950s to restrict population mobility between the rural and urban areas (4, 22). While the post-1978 economic reform has significantly altered this situation and promoted large-scale rural-to-urban migration, the rural migrants holding rural hukou (often referred to as *temporary migrants*) are not able to enjoy some urban public medical services, such as URBMI as the urban natives holding urban hukou (10, 12). Although there are institutionalized channels (e.g., the pursuit of higher education and employment in state-owned enterprises) for rural migrants to obtain urban hukou (these migrants are often regarded as *permanent migrants*) and thereby enjoy the same public medical services as the native urban residents, the proportion of these migrants is very low (22).

Alternatively, the temporary migrants without urban hukou can enroll in UEBMI that is funded by both the urban employers and migrant employees. However, as temporary rural migrants are often less educated and employed under informal and short-term contracts, the employers are less likely to pay insurance to the temporary migrants and prefer to contribute to insurance to more ‘qualified’ employees with more education and work experience (10, 12). Moreover, all the rural migrants are able to enroll in NRCMS, which aims to cover the residents holding rural hukou (12, 13). Recent studies (10–12), however, reveal that the temporary migrants without urban hukou may still have less access to NRCMS. This is partly due to the inflexible regulations of NRCMS, which require rural migrants to complete the registration in their home towns, and often involve a cumbersome procedure to reimburse medical bills from the urban hospitals (11, 23). This can be a potential barrier for the temporary migrants living in the cities to have access to public medical services. Generally, the hukou system, the design of insurances and the characteristics of temporary migrants are all the possible reasons for the low coverage of the insurance (14, 15).

### Research Objectives and Contributions

While the previous studies indicate a relatively low public medical insurance enrollment rate for the temporary rural migrants, most of these studies are limited to particular cities or provinces. Because of provincial differences within China in the terms of migration and public health policies (12), the findings of these studies may only reflect particular situations of those provinces and cannot be generalizable to the whole migrant population in China. Moreover, most previous research tends to treat migrants as a homogeneous group without distinguishing migrants at different migratory stages (i.e., temporary migrants without urban hukou and permanent migrants with urban hukou). A lack of such distinction does not allow us to explore the impact of hukou system on access of migrants to health resources.

This article aimed to compare the public and commercial medical insurance enrollment rates between the temporary, permanent migrants and urban natives on the national level, and discuss whether they have equal access to the health resources. *Thus, using a nationally representative sample, first contribution of this article is to compare public medical insurance enrollment rates between temporary, permanent migrants, and urban natives, while controlling for a wide arrange of demographic and socio-economic characteristics*.

Apart from these institutional barriers (e.g., hukou and the incomplete public medical system), the rural migrants overall have relatively low levels of income and awareness of disease prevention and insurance enrollment (4, 10, 13). For example, previous research shows that many migrant workers are not willing to enroll in medical insurance and try to avoid medical treatment even when they get ill to save on expense (10, 13). These factors might not only prevent the rural migrants from enrolling in public medical insurance, but also impede their enrollment in commercial insurance to a larger degree (10, 11). As commercial insurance often provides more flexible and diverse health services than public medical insurance, durable differences in commercial medical insurance enrollment may greatly intensify the health disparities between the rural migrants and urban natives. Although commercial medical insurance is of great importance to the health condition of residents, there is currently no research on China exploring the extent to which there is a gap between the rural migrants and urban natives in commercial medical insurance enrollment rates. *To fill this gap, the second contribution of this article is to compare the level of commercial medical insurance enrollment between temporary, permanent migrants and urban natives, while controlling for their different demographic and socio-economic characteristics*.

## Methods

### Data and Sample

The data used in this research come from the 2017 China General Social Survey (CGSS), which is one of the largest-scale cross-sectional surveys of China. The 2017 CGSS provides up-to-date information about medical insurance enrollment of the Chinese population. In the 2017 CGSS, a random multi-stage stratified probability-proportional-to-size (PPS) sampling was used to survey around 13,000 individuals. The multi-stage sampling procedure ensured that the sample is representative of the general population at province/municipality, street, and neighborhood level. Importantly, as the sampling procedure was based on the location of residence of respondents rather than household registration status, the sample is also representative of the whole migrant population in China. The data are available at http://cnsda.ruc.edu.cn/.

To construct the analytical sample, we first excluded those who reside in the rural areas (constitute approximately 36.08% of the original sample) because this study aimed to compare the medical insurance enrollment rates between the rural-to-urban migrants and native urban residents. The respondents who migrated from urban to rural areas due to its small sample sizes (approximately 3.9% of the original sample) were excluded from the study. After dropping a small number of observations with missing values (approximately 7.3%), this article obtained 7,398 valid cases for the models on public insurance enrollment and commercial insurance enrollment.

### Measures

#### Dependent Variables

The dependent variables are public and commercial medical insurance enrollment rates. The questionnaire asked respondents two questions about whether they are enrolled in public (such as UEBMI, URBMI, and NRCMS) and commercial medical insurance schemes. Both variables are binary variables (1 ‘Yes,’ and 0 ‘No’).

#### Migration Status

An important independent variable in this study is migration status. Specifically, we distinguish three population groups: urban natives (those who were born in the urban areas and have hukou), temporary migrants (rural migrants without urban hukou), and permanent migrants (rural migrants with urban hukou).

#### Other Covariates

Based on the ecological model of health behavior (24), we add other covariates into the model. The ecological models address the interactions and interdependences of people with sociocultural environments, especially the social determinants of health behaviors, which assist in providing a complete perspective and including requisite covariates. According to the theory, we choose individual factors, organizational factors, community factors, and public policy factors that were related to the commercial medical insurance enrollment rates. In terms of individual factors, as commercial insurance premium often depends on the demographic characteristics of people, we controlled for the age and gender of respondents, whether, or not, a respondent has a partner and children. Age was coded into the age groups, such as six categories: ‘18–25,’ ‘26–35,’ ‘36–45,’ ‘46–55,’ ‘55–65,’ and ‘66+.’ Moreover, as the health status of respondents is very likely to affect their willingness to participate into medical insurance, we controlled for a binary variable measuring the health status of respondents (1 ‘very good/good’ and 0 ‘fair/poor/very poor’).

In terms of the organizational factors and community factors, we controlled for several socio-economic characteristics, which are likely to affect the access of respondents to both public and commercial medical insurance. First, as the members of the Chinese Communist Party (CCP) generally have more access to a variety of socio-economic and medical resources, we controlled for CCP membership. In addition, we controlled for highest educational qualification of the respondents that included five categories: ‘no qualification,’ ‘primary school,’ ‘middle school,’ ‘high school,’ and ‘degree or above.’ Moreover, we controlled for annual household income of the respondents (measured by a quartile ranking) and their occupational status using Erikson-Goldthorpe-Portocarero (EGP) occupation codes, such as five occupational categories: ‘no work,’ ‘higher controller’ (EGP I, II, and V), ‘routine non-manual’ (EGP IIIa, IIIb, Iva, and IVb), ‘manual’ (EGP VI and VIIa), and ‘farm related work’ (EGP IVc and VIIb). In terms of public policy factors, we controlled for region that included three categories: West, Central, and East, as different regions of China have different migration and public policies.

### Analytical Strategy

As the dependent variables are binary, we used the weighted logistic regression models. Additionally, as objective of the research lies in comparing different models, the statistical analysis needs to consider the scaling problem, which refers to the potential bias that occurs when we compare odds ratios (*OR*s) across different models (25). This is because logistic regression estimates, which are rescaled based on a fixed residual variance, are likely to be affected by omitted variables (25). To avoid this problem, we follow Mood's suggestion to calculate the average marginal effect (AME) with a 95% *CI* (25). AME is interpreted as the change in the expected probability of the dependent variable with one unit increase of an independent variable. Specifically, we first compared the raw differences in the probability of enrolling in public and commercial medical insurance among the urban natives, temporary migrants, and permanent migrants, only controlling for region. Second, the demographic and socio-economic characteristics were added stepwise to the models to estimate the net differences. To adjust for the non-response and the complex survey design, appropriate sample weights provided by CGSS were used in the analysis.

## Results

### Descriptive Statistics

Table 1 reports descriptive statistics for the urban natives, temporary, and permanent migrants. Table 1 shows that while the temporary migrants have a lower public insurance enrollment rate (approximately 4.7% points) than the urban natives, the permanent migrants have a similar enrollment rate to urban natives. In terms of commercial insurance, the enrollment rates of temporary and permanent migrants are approximately 10 and 3.9% points lower than that of urban natives, respectively. Both the temporary and permanent migrants are more likely to live in east areas of China than in west and central areas as east areas are often the destination of rural migrants. In terms of the demographic characteristics, the permanent migrants are older, more likely to have children, and to be CCP members than the temporary migrants and urban natives. By contrast, the urban natives are slightly healthier, and have higher levels of education, income, and occupational status than both the temporary and permanent migrants. Although all the migrants are generally more socio-economically disadvantaged than the urban natives, the permanent migrants tend to fare better than the temporary migrants in terms of education, income, and occupational status.

**Table 1:** Descriptive statistics (column percentage).

	**Urban natives**	**Temporary** **migrants**	***T*** **-test**	**Permanent** **migrants**	***T*** **-test**
**Public medical insurance (Yes)**	93.7	89	−0.047***	93.1	−0.00600
**Commercial medical insurance (Yes)**	19.9	9.9	−0.101***	16	−0.039***
**Region**					
East	71.1	44.5	−0.265***	61.1	−0.100***
Central	14.6	24	0.094***	18.8	0.041***
West	14.3	31.4	0.171***	20.2	0.059***
**Female**	52.7	54	0.0130	54.6	0.0180
**Age groups**					
18–25	8.7	12.7	0.040***	3.6	−0.051***
26–35	15.9	19.6	0.036***	14.5	−0.0140
36–45	12.2	18.2	0.060***	21	0.089***
46–55	18.4	21.1	0.027**	20.1	0.0170
56–65	21.6	14.7	−0.069***	17.3	−0.043***
66+	23.3	13.8	−0.094***	23.5	0.00200
**Partnership (Yes)**	71.3	75.8	0.045***	82.7	0.115***
**Children (Yes)**	79.9	83	0.031***	92.3	0.124***
**Health status**					
Very healthy/healthy	59.2	59.6	−0.00400	56.6	0.026*
Fair/unhealthy/very unhealthy	40.8	40.4	0.00400	43.4	−0.026*
**CCP membership (Yes)**	18.7	4.5	−0.142***	21	0.023**
**Education levels**					
No qualification	3.1	13.1	0.100***	6.2	0.031***
Primary school	7.2	24.3	0.171***	15.2	0.080***
Middle school	23.7	32.8	0.091***	25.4	0.0170
High school	27.6	17.7	−0.100***	21	−0.066***
Degree or above	38.3	12.1	−0.262***	32.1	−0.062***
**Household income**					
The poorest	9.8	28.3	0.185***	13.1	0.033***
2^nd^ quartile	11.8	28.7	0.169***	18.9	0.071***
3^rd^ quartile	36.2	20.5	−0.157***	31	−0.052***
The richest	42.2	22.5	−0.197***	37	−0.052***
**EGP occupational status**					
No work	56.9	56.2	−0.00800	55.5	−0.0140
Higher controller	14.4	5.8	−0.086***	16.7	0.022**
Routine non-manual	10.4	8.2	−0.023***	8.2	−0.022**
Manual	16.5	16.1	−0.00400	16.3	−0.00200
Farm related work	1.7	13.7	0.120***	3.3	0.016***
**N**	3,124	2,445		1,829	

*t-tests are between the temporary migrants and urban natives, permanent migrants and urban natives, respectively*.

*** *p < 0.001*,

** *p < 0.01, and*

* *p < 0.05*.

## Regression Analysis

Table 2 reports AME of the weighted logistic regression models comparing public medical insurance enrollment rates between the urban natives and temporary and permanent migrants, respectively. Model 1 shows that without controlling for the demographic and socio-economic characteristics, the public medical insurance rate of temporary migrants is significantly lower (approximately 4.2% points) than that of urban natives. By contrast, the permanent migrants have a similar enrollment rate to urban natives. For the temporary migrants, Model 2 shows that after taking into account demographic characteristics, the enrollment rate of temporary migrants is still significantly lower (approximately 4.2% points) than that of urban natives, although the difference is slightly attenuated. In Model 2, the R squared increases from 1.08 to 2.42%, suggesting that the demographic characteristics overall play an important role in explaining public medical insurance enrollment.

**Table 2:** Average marginal effects (AME) of the logistic regression models predicting the public medical insurance enrollment rates.

	**Model 1**	**Model 2**	**Model 3**
**Migration status (ref = Urban natives)**			
Temporary migrants	−0.042***	−0.042***	−0.009
	(−0.061–−0.023)	(−0.062–−0.023)	(−0.030–0.012)
Permanent migrants	0.002	−0.006	0.000
	(−0.018–0.023)	(−0.027–0.015)	(−0.021–0.021)
**Region (ref = East)**			
Central	0.005	0.002	0.017
	(−0.016–0.025)	(−0.019–0.023)	(−0.004–0.039)
West	0.016	0.020*	0.037***
	(−0.004–0.037)	(−0.001–0.040)	(0.016–0.058)
**Demographic characteristics**		Y	Y
**Socio-economic characteristics**			Y
N	7,398	7,398	7,398
R squared	0.0108	0.0242	0.0615

*CIs in parentheses*,

*** * p < 0.001*,

** *p < 0.01, and*

* *p < 0.05. The demographic characteristics include the gender, age group, partnership, children, and health status of the household head. The control variables in the socio-economic characteristics are the Chinese Communist Party (CCP) membership, education level, household income, and Erikson-Goldthorpe-Portocarero (EGP) occupational class*.

Model 3 shows that after controlling for the socio-economic features, while the temporary migrants have no significantly lower level of public medical insurance enrollment than the urban natives, the difference reduces from 4.2% points to 0.9% points. This suggests that the disadvantaged socio-economic backgrounds of temporary migrants can greatly explain their lower public insurance enrollment rate. Moreover, in Model 3, the R squared increases from 2.42 to 6.15%, again suggesting that the socio-economic backgrounds are overall important for public medical insurance enrollment.

In terms of the commercial medical insurance enrollment rate, Model 1 in Table 3 shows that both the temporary and permanent migrants have significantly lower enrollment rates (approximately 12.6 and 5.6% points) than the urban natives. Moreover, people from central areas are less likely to enroll in commercial insurance than those from east areas. This is partly because east areas are more socio-economically developed and people from east areas have higher awareness of insurance enrollment. After taking into account the demographic characteristics, Model 2 shows that both the temporary and permanent migrants are still significantly less likely (approximately 14.1 and 6.4% points) to enroll in commercial medical insurance than the urban natives. The R squared increases from 3.85 to 7.90%, suggesting that the demographic characteristics play a fairly important role in explaining commercial medical insurance enrollment.

**Table 3:** Average marginal effects of the logistic regression models predicting the commercial medical insurance enrollment rates.

	**Model 1**	**Model 2**	**Model 3**
**Migration status (ref = Urban natives)**			
Temporary migrants	−0.126***	−0.141***	−0.075***
	(−0.153–−0.098)	(−0.169–−0.114)	(−0.105–−0.044)
Permanent migrants	−0.056***	−0.064***	−0.053***
	(−0.083–−0.030)	(−0.090–−0.038)	(−0.079–−0.027)
**Region (ref = East)**			
Central	−0.096***	−0.096***	−0.064***
	(−0.130–−0.063)	(−0.129–−0.064)	(−0.097–−0.031)
West	−0.078***	−0.085***	−0.058***
	(−0.108–−0.048)	(−0.115–−0.056)	(−0.088–−0.027)
**Demographic characteristics**		Y	Y
**Socio-economic characteristics**			Y
N	7,398	7,398	7,398
R squared	0.0385	0.0790	0.1183

*CIs in parentheses*,

*** *p < 0.001*,

** *p < 0.01, and*

* *p < 0.05. The demographic characteristics include the gender, age group, partnership, children, and health status of the household head. The control variables in socio-economic characteristics are CCP membership, education level, household income, and EGP occupational class*.

Finally, Model 3 shows that after controlling for socio-economic features, while the differences in commercial insurance enrollment rates are to some extent attenuated for the temporary migrants, both the temporary and permanent migrants have still significantly lower enrollments rates (approximately 7.5 and 5.3% points) than urban natives. In addition, it is worth noting that after controlling for these variables, the R squared increases from 7.90 to 11.83%, and the coefficients of many of the demographic variables are either attenuated or insignificant. This highlights the importance of socio-economic features in explaining commercial medical insurance enrollment.

## Discussion

Over the last several decades, large-scale internal population migration of China has stimulated heated discussion about the health condition of migrants and whether they have equal access to the health resources (21). While substantial research shows that the rural migrants fare worse than the urban natives in terms of indicators of infectious diseases (6, 7), occupational injuries (8), maternal and infant health (9), far less is known about equality of access, between the rural migrants and urban native residents, to public and commercial medical insurance. In addition, there is no research exploring commercial insurance enrollment of the rural migrants, and distinguishing migrants at different migratory stages. To fill these gaps, this article uses a nationally representative sample and compares the public and commercial medical insurance enrollment rates between urban natives and temporary, permanent rural migrants.

By analyzing and discussing the results of control variables, we can obtain some interesting findings. From the results above, respondent from the west and central regions of China are more likely to enroll in public insurance than those from east areas. This is partly because the temporary migrants who have less access to public insurance are more likely to reside in east areas. More specifically, the regression results show that those who are older and have a partner are more likely to enroll in public medical insurance than their counterparts. This is partly because those who are older and have a partner often have relatively stable jobs and are more concerned about their health condition. Additionally, those who have a better health condition are more likely to enroll in commercial medical insurance, highlighting the importance of medical insurance in promoting health.

In terms of public medical insurance, this article shows that while the permanent migrants have a similar enrollment rate to the urban natives, the temporary migrants have a significantly lower enrollment rate than the urban natives. The results show that the socio-economic characteristics can explain a large proportion of the difference, highlighting lower socio-economic backgrounds preventing the temporary migrants from gaining access to public medical insurance. This is consistent with previous research, highlighting the importance of employment and workplace characteristics in promoting the integration (26–28) and shaping health and life chances of the migrants (29–33). However, even after controlling for the demographic and socio-economic characteristics, there remains a persistent and significant difference in the enrollment rates between the temporary migrants and urban natives. This suggests that institutional barriers may still exist preventing the temporary migrants from gaining access to public medical insurance (21).

On the one hand, the temporary migrants without urban hukou cannot have access to some urban public medical services, such as URBMI. On the other hand, due to relatively low education level and unstable work contract of the temporary migrants, they are likely to suffer employer discrimination preventing them of gaining access to employee-based medical insurance, such as UEBMI. Moreover, as the temporary migrants live in cities and are a highly mobile population, they may also have less access to NRCMS, which aims to cover all the residents holding rural hukou (11, 12). This is partly due to many inflexible regulations of NRCMS, such as the regulation that stipulates that the migrants must return to their home towns to complete the registration of public insurance as well as a cumbersome procedure to reimburse medical bills from the urban hospitals (23). These regulations can be a significant institutional barrier preventing the temporary migrants from having access to NRCMS. In sum, while it is officially claimed that both URBMI and NRCMS can provide public medical insurance to all the residents of China regardless of hukou status, the temporary migrants are still a highly vulnerable group who have less access to public health resources (11, 12, 23). It is thus suggested that the government should adjust the rigid regulations and build a more comprehensive public medical insurance system to accommodate the diverse health needs of temporary migrants.

In terms of commercial medical insurance, both the temporary and permanent migrants have significantly lower enrollment rates than urban natives. The results show that the socio-economic features can to some extent explain the differences for temporary migrants, highlighting lower socio-economic backgrounds as a barrier preventing them from gaining access to commercial medical insurance (10, 13). As disadvantaged socio-economic status could impede the access to both public and commercial insurance, it is suggested that the host migrant cities could initiate training programs to help the rural migrants to integrate into the local labor market and improve their economic status.

Further results show that after controlling for the socio-economic features, the differences in commercial insurance enrollment rates remain significant for both the temporary and permanent migrants. The unexplained differences may be due to their lower health awareness as well as awareness of health insurance and how to enroll, as well as a lack of healthcare in neighborhoods and communities (10, 11, 13). This suggests that the reasons for the low levels of commercial insurance enrollment of migrants are complex and may result from the intersectionality of a variety of disadvantages (21).

However, this research has some limitations. First, the study design in the paper is observational, so no causal assumptions can be inferred, limiting the contribution to causal inference. Second, this article is limited to studying the differences in access to medical insurance between the rural migrants and urban natives without exploring in depth why certain rural migrants have less access to the medical resources. Understanding the complex causes of unequal access to health services is important for tackling durable health inequalities. Thus, future research could profitably extend the findings of this article by investigating the complex mechanisms of lower level of healthcare utilization of migrants and its potential implications for the health and migration policies of China.

## Conclusions

The paper uses a national sample, compares the public and commercial medical insurance enrollment rates between the urban natives and temporary, permanent rural migrants, finding both the temporary and permanent migrants have significantly lower commercial insurance enrollment rates compared with the urban natives. The results highlight significant institutional barriers preventing the temporary migrants from gaining access to public medical insurance and the adverse impact of disadvantaged socio-economic backgrounds on the access of temporary migrants to both public and commercial insurance.

## Data Availability Statement

Publicly available datasets were analyzed in this study. This data can be found here: http://cgss.ruc.edu.cn/.

## Author Contributions

SW has designed the research and conducted the original analyses. AL has re-conducted the analyses required by the reviewers and made significant contributions to the revision of the paper. WG has participated in the revision of the paper and made significant contributions to the literature review and framing of the paper. All authors contributed to the article and approved the submitted version.

## Funding

The research was funded by the Assistant Professor Start-up Grant and Center for Family and Population Research Faculty Development Grant at the National University of Singapore, the National Social Science Fund of China under Grant No. 20BRK040 and the National Natural Science Foundation of China under Grant No. 71921003.

## Conflict of Interest

The authors declare that the research was conducted in the absence of any commercial or financial relationships that could be construed as a potential conflict of interest.

## Publisher's Note

All claims expressed in this article are solely those of the authors and do not necessarily represent those of their affiliated organizations, or those of the publisher, the editors and the reviewers. Any product that may be evaluated in this article, or claim that may be made by its manufacturer, is not guaranteed or endorsed by the publisher.
